
JOURNAL INFORMATION
==============================
NLM Title Abbreviation: ScientificWorldJournal
Journal ID: ScientificWorldJournal
NLM Journal ID: 101131163
Journal ID: TSWJ

The Scientific World Journal

ISSN: 2356-6140
EISSN: 1537-744X
Publisher: Wiley

ARTICLE INFORMATION
==============================
PMCID: PMC6009234
PMID: 29973843
DOI: 10.1100/tsw.2002.66
Article ID: 161948
Article version: 1
Subjects: Short Report

A New Monoclonal Antibody Demonstrates That Muc4, the Intramembrane Ligand for Erbb2/Her2/Neu, Is Overexpressed in Multiple Carcinomas

Zhang Jin *
Carraway Kermit L.
Department of Cell Biology And Anatomy, University of Miami School of Medicine, P.O. Box 016960, Miami, Florida 33101, USA
*Jin Zhang: jzhang@med.miami.edu
Publication date: 2002
Electronic publication date: 2002 Jan 9
Volume: 2
First page: 140
Last page: 141
Copyright: Copyright © 2002 Jin Zhang and Kermit L. Carraway.
Copyright year: 2002
License: This is an open access article distributed under the Creative Commons Attribution License, which permits unrestricted use, distribution, and reproduction in any medium, provided the original work is properly cited.
License URL: https://creativecommons.org/licenses/by/3.0/

==============================
Advances in Gene Technology: The Genome and Beyond –
Structural Biology for Medicine (Proceedings of the 2002 Miami
*Nature Biotechnology* Winter Symposium)
*TheScientificWorld* 2002, 2(S2), 140–141
ISSN 1532-2246; DOI 10.1100/tsw.2002.66

### A NEW MONOCLONAL ANTIBODY DEMONSTRATES THAT MUC4, THE INTRAMEMBRANE LIGAND FOR ErbB2/HER2/Neu, IS OVEREXPRESSED IN MULTIPLE CARCINOMAS

Jin Zhang* and Kermit L. Carraway
Department of Cell Biology And Anatomy, University of Miami School of Medicine, PO Box 016960, Miami, Florida 33101
*jzhang@med.miami.edu

**INTRODUCTION.** MUC4, a membrane mucin, is expressed in multiple epithelia. Several studies have suggested that MUC4 gene is overexpressed in human carcinomas[1]. Human MUC4 was originally cloned and sequenced from airway cDNA library[2]. Most of the functional studies of MUC4 have been done with the rat homologue (SMC). Muc4 is a heterodimeric bifunctional glycoprotein and encoded on a single gene. It consists of a highly O-glycosylated glycoprotein, ascites sialoglycoprotein-1 (ASGP-1), which is tightly, but noncovalently linked to an N-glycosylated integral membrane glycoprotein ASGP-2[3]. Mucin ASGP-1 is proposed to have a protective role in epithelia. The transmembrane subunit ASGP-2 has two EGF-like domains, one of which acts as an intramembrane ligand for the receptor tyrosine kinase ErbB2/HER2/neu, which has been strongly implicated in cancer progression[4]. The MUC4 gene is expressed in numerous normal tissues including stomach, ovary, salivary gland, uterus, prostate, and thymus, thyroid, mammary, esophagus, small intestine, testis, and placenta. It is also found in secreted fluid, such as tears, milk, and saliva[5,6].

However, efforts to study MUC4 expression in cancer have been limited by the lack of antibodies that recognize MUC4 in formalin-fixed pathology samples. We have sought to address this problem by eliciting a new set of monoclonal antibodies. We report on one antibody that strongly stains some carcinomas, while not staining their normal tissue counterpart. These results suggest that this antibody may have value in diagnostic or prognostic studies of carcinomas.

**METHODS.** Purified ASGP-2 protein from 13672 rat mammary adenocarcinoma cells was injected into mice. Antisera from the mice were assayed against the antigen by Elisa and against the recombinant human ASGP-2 (expressed in Cos7 and HC11 cells) by immunoblotting to evaluate the titer. After cell fusion, the supernatants of hybridoma mass cultures were tested by Elisa and immunoblotting against the rat Muc4 and recombinant human ASGP-2 expressed in Cos7 and HC11 cells. Those supernatants that showed positive staining on both rat and human ASGP-2 were cloned and the supernatants from all clones generated were screened again. Cells of positive hybridoma clones were grown in a large volume for production of the antibody. Finally, the monoclonal antibody was affinity-purified using a Protein G affinity column (Amersham Pharmecia).

The specificity of the purified antibody for MUC4 was tested by immunoblotting against human milk in addition to the rat Muc4 and the recombinant human ASGP-2, and by immunoprecipitation on the recombinant human ASGP-2. Formalin-fixed and parafin-embedded tissues were stained with the antibody to analyze the expression of MUC4. All sections were observed under the microscope and the expression pattern was evaluated.

**RESULTS.** A hybridoma clone, designated as 1G8 monoclonal antibody, showed the highest OD reading on Elisa and the strongest staining on Western Blot against both rat and human ASGP-2. The affinity-purified antibody was tested on Western Blot against fresh human milk, a source of MUC4, in addition to the rat Muc4 and recombinant human ASGP-2. It strongly reacted with proteins from the milk. The primary band is about 140 Kda, corresponding in size to the recombinant human ASGP-2 and Muc4 transmembrane subunit from a number of rat tissues, such as the microvilli (MV). A few bands with higher Mr were also stained by 1G8 in both cell lysates of Cos7, HC11 and milk, suggesting the heterogeneity of the MUC4. 4F12, a rat ASGP-2 specific monoclonal antibody, was used as a control and did not react with any of the recombinant human ASGP-2. Similarly, the purified 1G8 mAb was also able to immunoprecipitate the recombinant human ASGP-2. Similar proteins to those stained in the straight Western Blot were detected in the precipitate by the 1G8 antibody blotting. More importantly, 1G8 strongly stains tissue array samples from several tumors, including lung, uterine and colon cancers, while not staining, or weakly staining, normal tissue regions from the same tumors.

**DISCUSSION**. This is the first reported anti-MUC4 monoclonal antibody, which strongly and specifically reacts with both rat and human ASGP-2. Most importantly, it stains formalin-fixed and paraffin-embedded human tissues. With the availability of tissue samples of different normal and cancer tissues, the analyses of MUC4 expression by 1G8 may be useful as a screening tool for assessing tumors for therapy or even as an antibody-directed therapy for selected cancers.

**REFERENCES**

1. Lopez-Ferrer, A., Curull, V., Barranco, C., Garrido, M., Lloreta, J., Real, F.X., and de Bolos, C. (2001) Am. J. Respir. Cell Mol. Biol. 24, 22–29.
2. Porchet, N., Nguyen, V.C., Dufosse, J., Audie, J.P., Guyonnet-Duperat, V., Gross, M.S., Denis, C., Degand, P., Bernheim, A., and Aubert, J.P. (1991) Biochem. Biophys. Res. Commun. 175, 414–422.
3. Carraway, K.L., Fregein, N.L., Carraway, C.A.C., and Carraway, K.L., III (1992) J. Cell Sci. 103, 299–307.
4. Carraway, K.L., III, Rossi, E.A., Komatsu, M., Price-Schiavi, S.A., Huang, D., Carvajal, M.E., Guy, P.M., Fregien, N.L., Carraway, C.A.C., and Carraway, K.L. (1999) J. Biochem. 274, 5263–5266.
5. Choudhury, A., Moniaux, N., Winpenny, J.P., Hollingsworth, M.A., Aubert, J.-P., and Batra, S.K. (2000) J. Biochem. 128, 233–243.
6. Rossi, E.A., McNeer, R.R., Price-Schiavi, S.A., Vanden Brande, J.M.H., Komatsu, M., Thompson, J.F., Carraway, C.A.C., Fregien, N.L., and Carraway, K.L. (1996) J. Biochem. 271, 33476–33485.
